# Development of ELISA based on *Bacillus anthracis* capsule biosynthesis protein CapA for naturally acquired antibodies against anthrax

**DOI:** 10.1371/journal.pone.0258317

**Published:** 2021-10-11

**Authors:** Tuvshinzaya Zorigt, Yoshikazu Furuta, Manyando Simbotwe, Akihiro Ochi, Mai Tsujinouchi, Misheck Shawa, Tomoko Shimizu, Norikazu Isoda, Jargalsaikhan Enkhtuya, Hideaki Higashi

**Affiliations:** 1 Division of Infection and Immunity, International Institute for Zoonosis Control (Former Research Center for Zoonosis Control), Hokkaido University, Sapporo, Japan; 2 Graduate School of Infectious Diseases, School of Veterinary Medicine, Hokkaido University, Sapporo, Japan; 3 Equine Research Institute, Japan Racing Association, Shimotsuke, Tochigi, Japan; 4 Laboratory of Microbiology, School of Veterinary Medicine, Hokkaido University, Sapporo, Japan; 5 Laboratory of Food Hygiene, Institute of Veterinary Medicine, Ulaanbaatar, Mongolia; USGS WCWRU: US Geological Survey Wisconsin Cooperative Wildlife Research Unit, UNITED STATES

## Abstract

Anthrax is a zoonotic disease caused by the gram-positive spore-forming bacterium *Bacillus anthracis*. Detecting naturally acquired antibodies against anthrax sublethal exposure in animals is essential for anthrax surveillance and effective control measures. Serological assays based on protective antigen (PA) of *B*. *anthracis* are mainly used for anthrax surveillance and vaccine evaluation. Although the assay is reliable, it is challenging to distinguish the naturally acquired antibodies from vaccine-induced immunity in animals because PA is cross-reactive to both antibodies. Although additional data on the vaccination history of animals could bypass this problem, such data are not readily accessible in many cases. In this study, we established a new enzyme-linked immunosorbent assay (ELISA) specific to antibodies against capsule biosynthesis protein CapA antigen of *B*. *anthracis*, which is non-cross-reactive to vaccine-induced antibodies in horses. Using *in silico* analyses, we screened coding sequences encoded on pXO2 plasmid, which is absent in the veterinary vaccine strain Sterne 34F2 but present in virulent strains of *B*. *anthracis*. Among the 8 selected antigen candidates, capsule biosynthesis protein CapA (GBAA_RS28240) and peptide ABC transporter substrate-binding protein (GBAA_RS28340) were detected by antibodies in infected horse sera. Of these, CapA has not yet been identified as immunoreactive in other studies to the best of our knowledge. Considering the protein solubility and specificity of *B*. *anthracis*, we prepared the C-terminus region of CapA, named CapA322, and developed CapA322-ELISA based on a horse model. Comparative analysis of the CapA322-ELISA and PAD1-ELISA (ELISA uses domain one of the PA) showed that CapA322-ELISA could detect anti-CapA antibodies in sera from infected horses but was non-reactive to sera from vaccinated horses. The CapA322-ELISA could contribute to the anthrax surveillance in endemic areas, and two immunoreactive proteins identified in this study could be additives to the improvement of current or future vaccine development.

## Introduction

Anthrax is a widely distributed zoonotic disease that occurs in every populated continent [1]. After the invention of an effective animal vaccine, outbreaks declined in many parts of the world; however, anthrax remains endemic in some regions of Africa and Asia [2–5]. Anthrax has a substantial economic and public health impact on countries with limited resources to develop anthrax control measures [6]. For example, Mongolia is a resource-limited country where anthrax is endemic, except the semi-desert and desert areas in the south [7].

Anthrax is caused by an encapsulated gram-positive spore-forming bacterium, *Bacillus anthracis*. *B*. *anthracis* infects a wide range of mammalians, including humans. Herbivores such as antelopes, buffaloes, cattle, sheep, goats, and horses are susceptible to anthrax, whereas birds and canids are comparatively resistant [8]. The infection occurs in herbivores through browsing, ingestion or inhalation of a high dose of spores from grazing lands; besides, carnivores are usually exposed through scavenging an infected animal carcass [9]. In addition, the role of tabanid flies and other blood-feeding insects in anthrax transmission in animals has been demonstrated [10, 11]. Humans often acquire anthrax infections from infected animals or materials contaminated with spores, such as wool, hide, and meat [12].

Two plasmids, pXO1 and pXO2, are essential for the virulence of *B*. *anthracis*. After ingestion or inhalation by the host, spores of *B*. *anthracis* germinate into vegetative cells that secrete the three pXO1-encoded toxin components: protective antigen (PA), edema factor (EF), and lethal factor (LF). PA is a host cell receptor-binding protein [13], EF is adenylate cyclase and a potent inhibitor of immune cell function [14], and LF cleaves mitogen-activated protein kinase and hinders cellular signaling pathways [15]. The pXO2 encodes genes involved in poly-γ-D-glutamic acid (γ-D-PGA), which protects the bacteria from the host phagocytic cells [16, 17]. A lack of either of the plasmids results in a significant loss of virulence of the bacterium [18]. The Sterne 34F2 strain, which lacks pXO2, still secretes the three major toxin components and retains immunogenicity with less virulence; thus, it is commonly used in anthrax veterinary vaccine production [19].

It was previously understood that anthrax mostly resulted in host death; however, field surveys in anthrax-endemic areas have suggested that herbivores infected with sublethal dose of *B*. *anthracis* spores could survive [20]. Furthermore, studies have indicated that exposure to a sublethal dose of spores likely elicits adaptive immune responses to *B*. *anthracis* [21, 22]. However, the effect of sublethal infection on the adaptive immune response of animals to anthrax is still poorly understood; scaled field studies are needed to detect naturally acquired antibodies in animals.

There is currently no serological test dedicated to distinguishing naturally acquired antibodies against *B*. *anthracis* from vaccine-induced immunity. The most of the available assays for anthrax serological diagnosis have been developed based on the PA of *B*. *anthracis* [23, 24]. Due to PA secretion from both naturally virulent and vaccine strains of *B*. *anthracis*, PA-based assays cannot distinguish the antibodies acquired by vaccination from those acquired by natural infection. So far, additional information such as the vaccination history of herds must differentiate the source of antibodies. However, while anthrax vaccination history is easily obtainable in countries with a good farm management system, accessing such data in developing countries is challenging because of poor record-keeping. For instance, herders in Mongolia have nomadic pastoralism where households migrate to various places to seek pastures for their livestock. Such movements often lead to the loss of important animal records, including vaccination history. In addition, animal identification systems in most local communities are largely uncoordinated, thus complicating the discrimination of vaccinated animals from unvaccinated animals.

Herein, we developed a new enzyme-linked immunosorbent assay (ELISA) test for detecting antibodies against capsule biosynthesis protein CapA of *B*. *anthracis*, which is non-cross-reactive to vaccine-induced antibodies. We screened genes on the pXO2 for ELISA antigen candidates because of the differences between virulent *B*. *anthracis* (pXO1^+^, pXO2^+^) and anthrax vaccine strains (pXO1^+^, pXO2^−^). Further, we identified that capsule biosynthesis protein CapA (GBAA_RS28240) and peptide ABC transporter substrate-binding protein (GBAA_RS28340) are immunoreactive to hyperimmune horse anti-*B*. *anthracis* serum. We also found that the C-terminus region of CapA, named CapA322, is soluble and specific to *B*. *anthracis*. Therefore, we developed the CapA322-ELISA using the antigen.

## Materials and methods

### Horse test sera

Hyperimmunized antiserum of horses infected with virulent *B*. *anthracis* Pasteur No. 1 strain (pXO1^+^, pXO2^+^), named PC1 in this study (also known as the Ascoli serum), was obtained from the National Institute of Animal Health, Japan.

Two naturally infected horse sera (PC2 and PC3) were provided by the Institute of Veterinary Medicine of Mongolia. According to records, the two horses showed clinical anthrax symptoms, and *B*. *anthracis* was isolated from the nasal discharge samples and confirmed with polymerase chain reaction (PCR) [25, 26].

To prepare serum samples of vaccinated horses, four 2-year-old female horses (Vac_H1 to Vac_H4) were subcutaneously injected with Sterne vaccine containing 2 × 10^6^ spores/ml, as recommended by the manufacturer (KM Biologics, Japan). Serum samples were collected before and after vaccination. After vaccination, serum samples were collected every three days starting from day 3 to day 45 postvaccination. Later, samples were obtained every seven days until day 56 postvaccination. The antibody response to PA of *B*. *anthracis* was evaluated by PAD1-ELISA [27]. Serum samples with high anti-PA-D1 immunoglobulin G (IgG) titers were obtained on day 21 postvaccination (named Vac_H1D21–Vac_H4D21 for the four horses, respectively) and were used to test the cross-reactivity of CapA322-ELISA.

Two naive horse sera (NC1 and NC2) were purchased from Invitrogen, USA, and KOHJIN BIO, Japan, to serve as negative controls.

### Ethical statement

Ethical clearance and research approvals were obtained from the Animal Experiment Committee of Equine Research Institute, Japan Racing Association (Reference: 20–32), and immunization and sampling were conducted according to approved protocols.

### *In silico* analyses

All coding sequences (CDSs) on pXO2 of *B*. *anthracis* Ames ancestor strain (GenBank accession number: AE017335) were obtained from the National Center for Biotechnology Information and analyzed to predict their cellular localization, secretion, and functional domains. PSORT was used to predict cellular protein localization [28], and SignalP was used to predict cleavable N-terminus signal peptide regions [29]. Lipoprotein signal peptides identified by LipoP [30], and membrane-associated proteins with transmembrane helix were predicted by the transmembrane-hidden Markov model (TMHMM) algorithm [31]. The domains and active sites of the proteins were identified by PROSITE [32]. The criteria for selecting candidate genes focused on secreted and surface-exposed CDS products. All CDSs were scored based on the presence of the predicted signal peptide, lipoprotein signal, TMHMM domain, putative domain information, and localization on the cell surface or extracellular secretion. Eight CDSs that scored three or more were selected (S1 Table).

### Construction of strains and plasmids

Primers for amplification of candidate CDSs were designed and synthesized by Integrated DNA Technologies (Table 1). Candidate CDSs were amplified from genomic DNA of *B*. *anthracis* CZC5 [33] using KOD FX Neo (TOYOBO, Japan). The vector F (5′-GGGTCGACTCGAGCGGCCGCA-3′) and vector R (5′-GGATCCCAGGGGCCCCTGGAACAG-3′) were used for the linearization of pGEX-6P-2 plasmid, which expresses the glutathione S-transferase (GST) fusion protein. The amplified genes were incorporated into the plasmid using Gibson Assembly [34]. The amplified PCR products were analyzed on 1% agarose gel and extracted using the QIAquick Gel Extraction Kit (Qiagen, Germany). The purified fragments with 5′ complementary overhangs were combined in a 1:2 molar ratio of the vector, and the fragments were inserted with 20 μl of Gibson Assembly Master Mix (New England Biolabs, MA) and additional nuclease-free water to obtain a reaction volume of 40 μl. The reaction was then performed at 50°C for 15 min, and the assembled constructs were desalted for an hour using MF-Millipore membrane filter 0.025 μm (Merck, Germany) on distilled water. *Escherichia coli* 10β (New England Biolabs, MA) was transformed with each construct through electroporation. The cells and constructs were mixed and transferred to the Bio-Rad 0.1 cm gap Gene Pulser cuvettes. Electroporation was performed using a Gene Pulser Xcell Electroporator (Bio-Rad, CA) set to 1,800 V, 25 μF, and 200 Ω. After electroporation, the cells were immediately transferred to 1 ml of lysogeny broth (LB) and incubated at 37°C for an hour at 180 rpm. Next, 200 μl of the culture was plated on LB agar supplemented with 50 μg/ml ampicillin for selection. The DNA from constructs was purified using a QIAprep Spin Miniprep Kit (Qiagen, Germany), and the sequence of each gene of interest was confirmed by Sanger sequencing using a 3130 xl Genetic Analyzer (Applied Biosystems, MA).

**Table 1:** Primer list.

Primer name	Locus tag	Primer sequence
TZ_F006	GBAA_RS28005	5’-CTGTTCCAGGGGCCCCTGGGATCCATGGCAGCTACACAAGAAACAGCC-3’
TZ_R006		3’-TGCGGCCGCTCGAGTCGACCCTCATCTTGGTACTCTTCGAATTCCTG-5’
TZ_F012	GBAA_RS28035	5’-CTGTTCCAGGGGCCCCTGGGATCCATGGCTACTATGAAAATAAAAGAATGG-3’
TZ_R012		3’-TGCGGCCGCTCGAGTCGACCCTTATCTTCTACGCAATTGATCTGTCC-5’
TZ_F029	GBAA_RS28110	5’-CTGTTCCAGGGGCCCCTGGGATCCATGTGTAAAAGGTTTAAGTTTTTATTGGCTG-3’
TZ_R029		3’-TGCGGCCGCTCGAGTCGACCCTTAATTTGTTTTCTTAAATATATTTTGTTTATTAACG-5’
TZ_F043	GBAA_RS28165	5’-CTGTTCCAGGGGCCCCTGGGATCCATGAACACTAAGGGAATTATAGCAAAAC-3’
TZ_R043		3’-TGCGGCCGCTCGAGTCGACCCTTAGTAATAAGCAGACATGTTATGACCTTTC-5’
TZ_F060	GBAA_RS28240	5’-CTGTTCCAGGGGCCCCTGGGATCCATGAGACGAAAATTGACATTTCAAG-3’
TZ_R060		3’-TGCGGCCGCTCGAGTCGACCCTCAAGTTGTTGTCTCCACTGATAC-5’
TZ_F068	GBAA_RS28275	5’-CTGTTCCAGGGGCCCCTGGGATCCATGAAAATAATAAAATTGTTGATTACATATGG-3’
TZ_R068		3’-TGCGGCCGCTCGAGTCGACCCTATTTAGAAATTACTGTAGCTAGAACACGTTCG-5’
TZ_F083	GBAA_RS28340	5’-CTGTTCCAGGGGCCCCTGGGATCCATGTTAAAAAAAGTAACGCCTATTGTGG-3’
TZ_R083		3’-TGCGGCCGCTCGAGTCGACCCTTATTTCTTCACTTCAGTCCACTTATAG-5’
TZ_F100	GBAA_RS28430	5’-CTGTTCCAGGGGCCCCTGGGATCCATGAAGTATAAAACGCATCTTACAACAAG-3’
TZ_R100		3’-TGCGGCCGCTCGAGTCGACCCTTAACTAAATAACGCTTTAAAGGATTCTAAAAT-5’
TZ_F322	capA322	5’-CTGTTCCAGGGGCCCCTGGGATCCCGTGATAATGGTACTGCAATTCTTG-3’
TZ_R322		3’-TGCGGCCGCTCGAGTCGACCCTCAAGTTGTTGTCTCCACTGATAC-5’

### Protein expression and confirmation of immunogenicity

*E*. *coli* BL21 cells were transformed with each construct for protein expression. The transformed *E*. *coli* BL21 cells were grown in 3 ml LB supplemented with 50 μg/ml of ampicillin at 37°C for 18 h at 180 rpm. The OD_600_ of the cultures was adjusted to 0.05 in 3 ml LB or terrific broth (TB) with 50 μg/ml of ampicillin, and the cells were grown at 37°C at 180 rpm. When the OD_600_ reached 0.8, the expression of proteins was induced by adding isopropyl β-D-thiogalactopyranoside (IPTG) to a final concentration of 0.2 mM. The cells were grown at 37°C for 4 h at 180 rpm. In addition, uninduced cell cultures were prepared and used as controls. The cells were harvested at 8,000 rpm for 10 min and washed twice with phosphate-buffered saline (PBS). The cell pellets were resuspended in 250 μl of lysis buffer (PBS containing 0.05% Tween-20, 1 mM PMSF, 0.1 mM benzamidine, pH 7.5) and lysed using a Branson 450 Analog Sonifier (Branson Ultrasonics). The total lysate, supernatant, and pellet fractions of cells were collected. Then, 15 μl of each collected fraction was diluted in 5 μl of sodium dodecyl sulfate (SDS) gel-loading buffer (50mM Tris-HCl pH 6.8, 2% SDS, 10% glycerol, 1% beta-mercaptoethanol, 12.5 mM EDTA, 0.02% bromophenol blue), and 5 μl of diluted sample was analyzed using 10% SDS polyacrylamide gel electrophoresis (SDS-PAGE).

Detection of expressed GST fusion proteins was achieved by Western blotting using GST Mouse Monoclonal IgG (Santa Cruz, USA) and anti-mouse IgG-HRP (GE Healthcare UK Limited). To identify which proteins were immunoreactive, the hyperimmunized antiserum of horses (PC1) infected with virulent *B*. *anthracis* strain was used to probe the proteins in the cell pellet fraction, followed by probing with anti-horse IgG (Jackson ImmunoResearch Laboratories, Inc, Pennsylvania, USA) for detection. Upon visualization of the Western blotting, signals of target proteins higher than the background were selected as positive signals.

### Expression and purification of CapA322

We defined the peptide derived from the 322^nd^ to the 411^th^ amino acid residues of capsule biosynthesis protein CapA as CapA322. Primers for amplifying partial coding sequence of CapA are described in Table 1, and pGEX-6P-2 was used to construct pTZ006. The protein expression in *E*. *coli* was conducted using the method described above. After the expression process, the cells were harvested and lysed using the Branson 450 Analog Sonifier (Branson Ultrasonics). The resulting suspension was centrifuged at 4°C for 15 min at 15,000 rpm. From the supernatant, GST-tagged recombinant CapA322 was purified using Glutathione Sepharose 4 beads (GE Healthcare) according to a batch protocol of the manufacturer. The GST tag was cleaved from the protein using the PreScission Protease (GE Healthcare) at 4°C for 18 h. Then, recombinant CapA322 was eluted with an elution buffer (50 mM Tris, 150 mM NaCl, 1 mM EDTA, 1 mM DTT, pH 7.5). The elution buffer was changed to 25 mM 2-(N-morpholino) ethane sulfonic acid (MES), pH 6.5, using Amicon Ultra centrifugal device (Amicon Ultra). After buffer exchange, the collected fraction was loaded onto a Resource-S column (GE Healthcare) equilibrated with 25 mM MES, pH 6.5. A NaCl gradient from 0 mM to 150 mM was used for elution. The eluted CapA322 protein was stored in aliquots at −80°C. Fractions at all steps of the expression and purification processes were analyzed using 10% SDS-PAGE and Western blotting. For the Western blotting analyses of CapA322, PC1 and NC1 sera were used as primary antibodies, whereas anti-horse IgG-HRP (Jackson ImmunoResearch Laboratories, Inc, Pennsylvania, USA) was used as a secondary antibody.

### CapA322 in-house ELISA

Checkerboard titration were performed to determine the optimal concentration of the reagents [35]. Flat-bottomed 96-well microtiter plates (Corning) were coated with 100 μl per well of serially diluted CapA322 in coating buffer (0.1 M NaHCO_3,_ pH 9.6) and incubated overnight at 4°C. The antigen-coated plates were washed three times with 300 μl wash buffer (PBS containing 0.05% Tween-20, pH 7.4), and the wells were blocked with 100 μl blocking buffer (3% skim milk in PBS containing 0.05% Tween-20, pH 7.4) for 1 h at room temperature (RT). After being briefly washed three times, the sera of PC1 and NC1 were each diluted from 1:100 to 1:6400 in a blocking buffer. Then, 100 μl of the diluted solution was added to wells in triplicate and incubated at RT for 1 h. After being washed again, goat anti-horse IgG-HRP (Sigma-Aldrich) was diluted to 1:15,000 in blocking buffer, and 100 μl of the diluted antibody solution was added to the wells and incubated at RT for 1 h. After washing, an antigen and antibody complex was detected by adding 100 μl 3,3′,5,5′-tetramethylbenzidine substrate (KPL) per well. The reaction was stopped by adding 50 μl 1N HCl per well followed by 20 min incubation at RT. Absorbance at 450 nm (OD 450) was measured using a microtiter plate reader (Thermo Scientific, USA).

### Statistical analysis

Each horse serum sample was tested in technical triplicate by PAD1-ELISA and CapA322-ELISA. One sample (Vac_H4D21) did not show the expected antibody response to vaccination when analyzed by PAD1-ELISA, suggesting the failure of immunization. Therefore, this sample was excluded from the following statistical analysis. The rest of the samples were categorized as positive group (PC; n = 3), negative group (NC; n = 2), and vaccinated group (Vac; n = 3). Then, we calculated the relative OD values of serum samples by dividing OD values obtained from PAD1-ELISA by those from CapA322-ELISA. The one-way ANOVA with Tukey’s multiple comparison test was conducted to test for differences among the groups.

## Results

### Screening of CDS-encoding secreted and surface-associated proteins on pXO2 plasmid

To select potential antigen candidates among 105 CDSs encoded on pXO2 of *B*. *anthracis* Ames ancestor strain, we conducted *in silico* screening. We identified functional features that could make proteins more antigenic by searching for anchoring domain, secretion signal peptide, and cellular localization [36, 37].

We used 5 informatics tools for the screening. Signal P [29] predicted the product of 12 CDSs to possess the N-terminus signal peptides and secreted through the classical Sec pathway. PSORT [28] predicted 2 extracellular proteins, 3 cell wall-associated proteins, 27 cell membrane-related proteins, and 35 cytoplasmic proteins. LipoP [30] predicted 24 proteins: 7 with putative Sec signal peptide SpI sites, 2 with lipoprotein signal peptides SpII sites, and 15 with transmembrane helix domain. TMHMM [31] predicted 33 proteins, including one to six transmembrane helix domains. In addition, PROSITE [32] indicated that several proteins have S-layer homology domains, papain-like cell wall hydrolase domain (NLPC/60), membrane lipoprotein lipid attachment sites, and serine lysine active sites (S1 Table).

We focused mainly on secreted and surface-exposed CDS products that are considered relevant for identifying targets for eliciting protective immunity. Combining the results of the 5 tools, we selected 8 CDSs based on predicted signal sequence, transmembrane helix domain, and cellular localization, considering potential exposure to a host cell and probability to generate host protective response. Our candidate proteins were predicted to be secreted, and surface-associated proteins were observed to have 0–5 transmembrane segments (Table 2).

**Table 2:** *In silico* selected CDSs for identification of immunoreactivity.

Locus tag number	Product name	*In silico* data				
		Cellular localization	Signal peptide^a^	Lipoprotein signal peptide^a^	TMHMM number^b^	Domains and active sites
GBAA_RS28005	Lysozyme	Extra cellular	+	-	1	NlpC/P60
GBAA_RS28035	Hypothetical protein	Membrane	+	+	5	
GBAA_RS28110	Hypothetical protein	Membrane	+	-	1	PROKAR Lipoprotein
GBAA_RS28165	Amidase	Cell wall	+	-	0	SLH
GBAA_RS28240	Capsule biosynthesis protein capA	Membrane	+	-	1	
GBAA_RS28275	Signal peptidase	Membrane	+	-	1	Serine, lysine active sites
GBAA_RS28340	Peptide ABC transport substrate binding protein	Cell wall	+	+	0	PROKAR Lipoprotein
GBAA_RS28430	Metal dependent hydrolase	Membrane	+	-	3	

^a^If secretion or lipoprotein signals were detected; +, If not -.

^b^Transmembrane helix domains.

### Expression and immunoreactivity of candidate proteins

**Fig 1:**
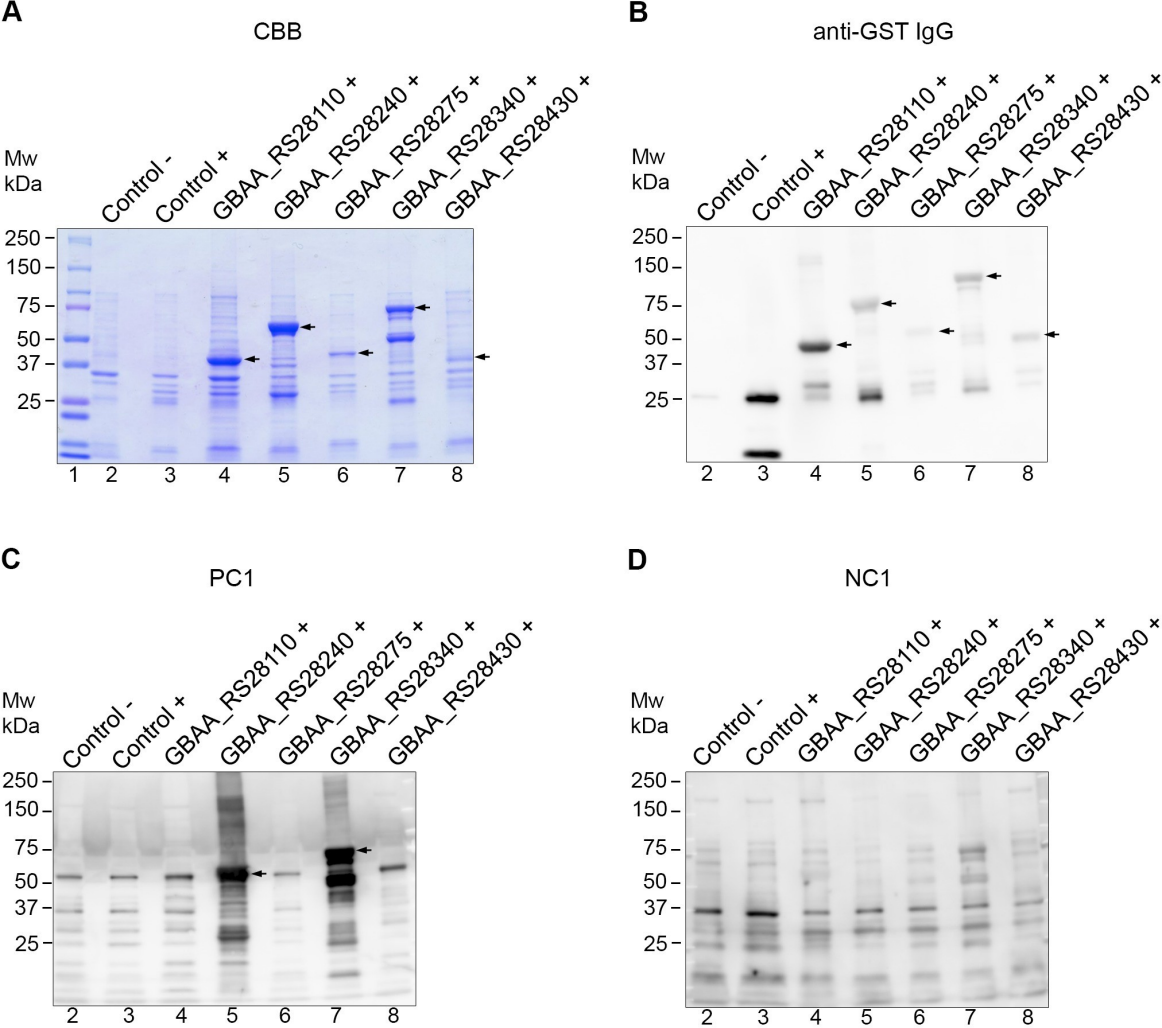
Expression and immunoreactivity of candidate proteins. (A) Coomassie brilliant blue (CBB) staining and (B–D) Western blotting of the proteins in cell pellet fractions of the control and candidate protein-expressing *Escherichia coli* strains grown in terrific broth. In Western blotting, the proteins in the cell pellets were probed with different antibodies: (B) anti-glutathione S-transferase (GST) immunoglobulin G; (C) horse hyperimmunized anti-*Bacillus anthracis* serum (PC1); (D) naive horse serum (NC1). Lane 1, Mw, molecular weight marker (in kDa); lane 2, control strain which is *E*. *coli* BL21 harboring empty pGEX-6P-2 without IPTG induction; lane 3, control strain which is *E*. *coli* BL21 harboring empty pGEX-6P-2 with 0.2 mM IPTG induction; lane 4, *E*. *coli* BTZ001 expressing recombinant hypothetical protein (GST-GBAA_RS28110: 44 kDa); lane 5, *E*. *coli* BTZ002 expressing recombinant capsule biosynthesis protein CapA (GST-GBAA_RS28240: 72 kDa); lane 6, *E*. *coli* BTZ003 expressing recombinant signal peptidase (GST-GBAA_RS28275: 47 kDa); lane 7, *E*. *coli* BTZ004 expressing recombinant peptide ABC substrate-binding protein (GST-GBAA_RS28340: 84 kDa); lane 8, *E*. *coli* BTZ005 expressing recombinant metal-dependent hydrolase (GST-GBAA_RS28430: 46 kDa). The arrows indicate target proteins in the expected sizes.

To further identify whether candidate proteins were immunoreactive, the proteins were probed with the hyperimmunized antiserum of horses infected with virulent *B*. *anthracis* (PC1). CDSs were cloned and expressed in *E*. *coli*. We successfully constructed *E*. *coli* strains that express our candidate proteins fused with GST tag at the N-terminus for 5 of 8 CDSs selected from *in silico* analyses (Table 3). The size of the target proteins was verified using SDS-PAGE (Fig 1A) and Western blotting with antibodies against the GST tag (Fig 1B). The target proteins at the expected molecular sizes were visualized in the lysate, supernatant or pellet fraction of the cell lysate, indicating soluble or insoluble expression (S1 Fig). After expression verification, proteins were probed with hyperimmune antiserum from horses infected with virulent *B*. *anthracis* (PC1) to identify their immunoreactivity. Of the 5 expressed proteins, only the capsule biosynthesis protein CapA (GBAA_RS28240) and peptide ABC transporter substrate-binding protein (GBAA_ RS28340) reacted with PC1 serum (Fig 1C). Serum from the naive horse (NC1) showed only background signal with the screened proteins, indicating that the proteins detected using the PC1 serum resulted from an antibody response induced by fully virulent *B*. *anthracis* infection (Fig 1D).

**Table 3:** Constructs and strain list.

Strain or plasmid	Description	Reference
Plasmids		
pGEX-6P-2	Cloning vector	[38]
pTZ001	pGEX-6P-2 cloned with GBAA_RS28110	This study
pTZ002	pGEX-6P-2 cloned with GBAA_RS28240	This study
pTZ003	pGEX-6P-2 cloned with GBAA_RS28275	This study
pTZ004	pGEX-6P-2 cloned with GBAA_RS28340	This study
pTZ005	pGEX-6P-2 cloned with GBAA_RS28430	This study
pTZ006	pGEX-6P-2 cloned with C-terminus region of GBAA_RS28240	This study
Strains		
*E*. *coli*		
BL21	B F^– ^*ompT gal dcm lon hsdS*_B_(r_B_^–^m_B_^–^) [malB^+^] _K-12_(λ^S^)	[39]
BTZ001	BL21 harboring pTZ001	This study
BTZ002	BL21 harboring pTZ002	This study
BTZ003	BL21 harboring pTZ003	This study
BTZ004	BL21 harboring pTZ004	This study
BTZ005	BL21 harboring pTZ005	This study
BTZ006	BL21 harboring pTZ006	This study

### Evaluation of CapA322 as ELISA antigen

We analyzed the sequence specificity of the two proteins, CapA (GBAA_RS28240) and peptide ABC transporter substrate-binding protein (GBAA_RS28340), that showed immunoreactivity to PC1. When the entire length of the proteins was analyzed with tBLASTn, the peptide ABC transporter substrate-binding protein showed high sequence similarity with a protein encoded in *Bacillus cereus*. Alternatively, the CapA showed low sequence similarity to common *B*. *cereus* strains (~56%) and other *Bacillus* species (55%–60%) evolutionarily related to *B*. *anthracis*. A few unusual *B*. *cereus* strains possess virulent plasmids similar (85%–89%) to those of *B*. *anthracis*, including the toxin and capsule-coding genes (Table 4).

**Table 4:** Sequence similarity of two immunoreactive proteins identified by Western blotting with horse hyperimmune antisera and CapA322.

Whole length CapA						
ID	Species	Similarity	Positive	Coverage	Plasmid	Chromosome
CP020941.1	*B*. *cereus* strain BC-AK plasmid pBCXO2	89%	100%	100%	+	
CP001748.1	*B*. *cereus* biovar anthracis str. CI plasmid pCI-XO2	89%	100%	100%	+	
CP009317.1	*B*. *cereus* 03BB102 plasmid	86%	96%	94.10%	+	
CP001406.1	*B*. *cereus* 03BB102 plasmid p03BB102_179	86%	96%	94.10%	+	
CP009636.1	*B*. *cereus* 03BB108 plasmid pBFI_2	85%	94%	94.10%	+	
CP017574.1	*B*. *thuringiensis strain* SCG04-02 plasmid PSCG. . .	57%	79%	93.70%	+	
CP015177.1	*B*. *thuringiensis serovar* alesti strain BGSC 4. . .	60%	79%	87.30%	+	
CP023179.1	*B*. *cereus* strain CC-1	56%	79%	93.60%		+
CP030926.1	*B*. *butanolivorans* strain PHB-7a	55%	76%	93.90%		+
CP017080.1	*B*. *muralis* strain G25-68	55%	76%	93.90%		+
CapA322						
ID	Species	Similarity	Positive	Coverage	Plasmid	Chromosome
CP020941.1	*B*. *cereus* strain BC-AK plasmid pBCXO2	81%	100%	100%	+	
CP001748.1	*B*. *cereus* biovar anthracis str. CI plasmid pCI-XO2	81%	100%	100%	+	
CP009317.1	*B*. *cereus* 03BB102 plasmid	91%	95%	73.30%	+	
CP001406.1	*B*. *cereus* 03BB102 plasmid p03BB102_179	91%	95%	73.30%	+	
CP009636.1	*B*. *cereus* 03BB108 plasmid pBFI_2	86%	89%	73.30%	+	
CP017704.1	*B*. *simplex* NBRC 15720 = DSM 1321	59%	86%	70%		+
CP011008.1	*B*. *simplex* strain SH-B26	60%	84%	70%		+
CP030063.1	[*Brevibacterium] frigoritolerans* s*train ZB201705*	57%	84%	70%		+
CP030926.1	*B*. *butanolivorans* strain PHB-7a	56%	83%	70%		+
CP017080.1	*B*. *muralis* strain G25-68	57%	76%	70%		+
Peptide ABC substrate-binding protein						
ID	Species	Similarity	Positive	Coverage	Plasmid	Chromosome
CP001748.1	*B*. *cereus biovar* anthracis str. CI plasmid pCI-XO2	93%	95%	100%	+	
CP020941.1	*B*. *cereus* strain BC-AK plasmid pBCXO2 sequence	90%	94%	100%	+	
CP015180.1	*B*. *thuringiensis* serovar alesti strain BGSC 4	90%	94%	100%	+	
DQ025752.1	*B*. *thuringiensis* serovar kurstaki plasmid pAW	90%	94%	100%	+	
CP018742.1	*B*. *cereus* strain FORC_047 plasmid pFORC47_2	90%	94%	100%	+	
CP003691.1	*B*. *thuringiensis* MC28 plasmid pMC183	87%	92%	100%	+	
CP024687.1	*B*. *wiedmannii* bv. thuringiensis strain FCC41	86%	93%	100%	+	
MG710485.1	*B*. *thuringiensis* serovar israelensis strain B	86%	92%	100%	+	
CP015154.1	*B*. *thuringiensis* strain Bc601 plasmid pBTBC4	86%	92%	100%	+	
CP013059.1	*B*. *thuringiensis* strain YWC2-8 plasmid pYWC2-	86%	92%	100%	+	

**Fig 2:**
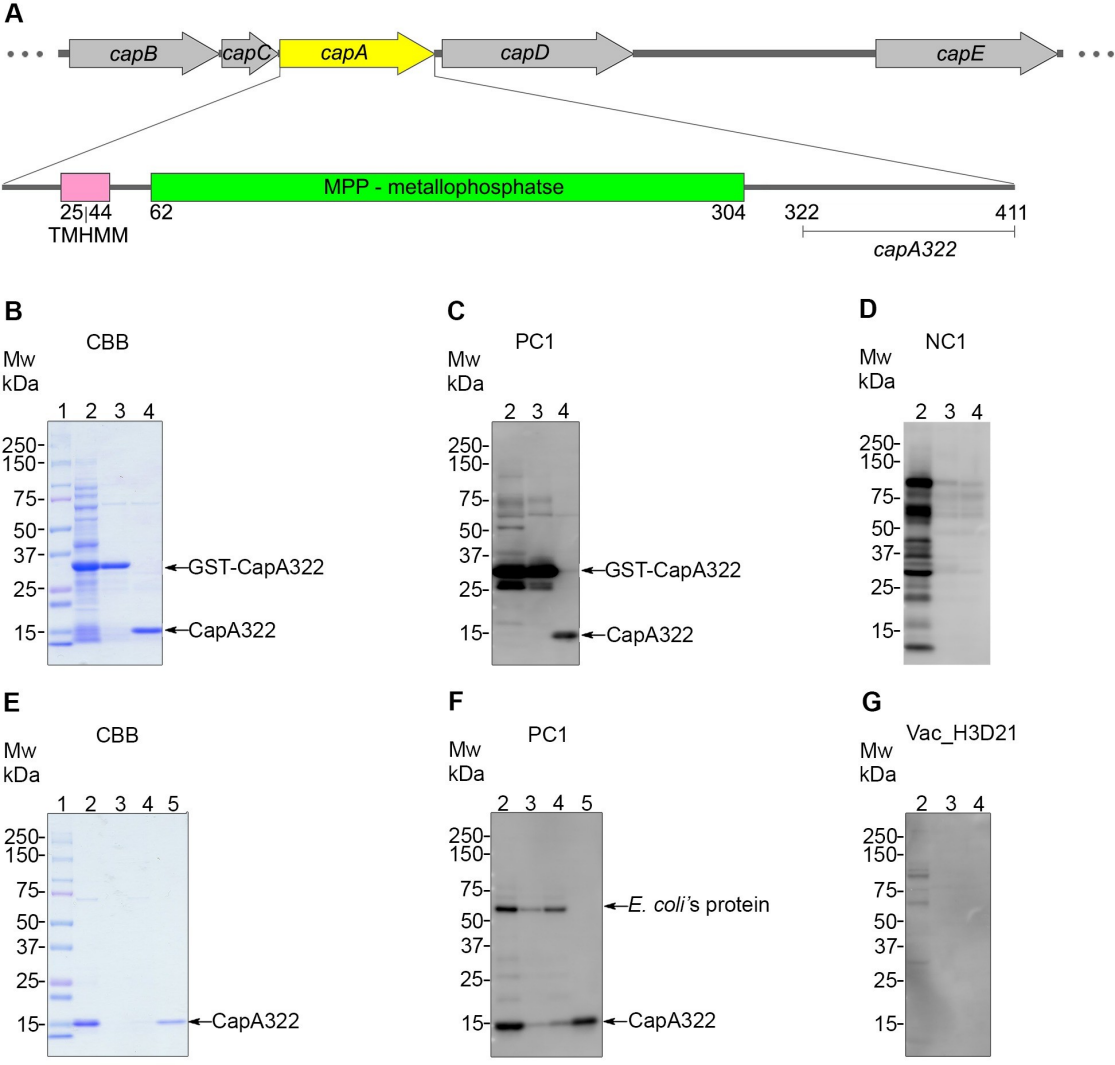
Expression and purification of CapA322. (A) Genetic map of *Bacillus anthracis* capsule-coding operon revealing the CapA322, C-terminus region (amino-acid 322–411) of CapA used in antigen preparation. (B) Coomassie brilliant blue staining (CBB) and (C, D, and G) Western blotting of the fractions collected after affinity batch purification. In Western blotting, proteins were probed with different horse sera: (C) horse hyperimmunized anti-*B*. *anthracis* serum (PC1); (D) naive horse serum (NC1); (G) vaccinated horse serum (Vac_H3D21). Lane 1, Mw, molecular weight marker (in kDa); lane 2, the supernatant fraction applied to affinity beads (GST-CapA322: 37 kDa); lane 3, beads bound; lane 4, elute after treatment with PreScission Protease (CapA322: 11 kDa). (E and F) CBB and Western blotting of the fractions collected during the cation exchange process. Lane 1, Mw, molecular weight marker (in kDa); lane 2, sample loaded onto the cation exchange chromatography column; lanes 3 and 4 flow-throughs; lane 5, the eluted protein. Host cell-derived contaminants indicated as *Escherichia coli*’s protein.

Considering the low solubility of whole length CapA, we selected its soluble region at the C-terminus from 322^nd^ to 411^th^ (Fig 2A). This region also showed even lower sequence similarity (55%) with homologs in common *B*. *cereus* strains than the N-terminus region. Thus, we tested if the CapA peptide of this region, defined as CapA322, shows better solubility and retains immunoreactivity. The CapA322 was expressed as a GST fusion protein in *E*. *coli* and solubilized in the supernatant fraction of the cell lysate. The protein size was verified using SDS-PAGE and Western blotting with anti-GST IgG (S2 Fig). In GST-tag affinity purification before and after GST-tag cleavage, a GST-CapA322 band of approximately 37 kDa and CapA322 of 11 kDa were detected (Fig 2B). The CapA322 reacted with hyperimmune antiserum from horses infected with virulent *B*. *anthracis* (PC1) but did not react with serum from the naive horse (NC1) (Fig 2C and 2D). After the first purification process, the remaining host cell derivative contaminants were removed using an ion-exchange chromatography column (Fig 2E and 2F). From a 1L culture, approximately 0.6 mg of CapA322 was obtained with a purity of >90%. As expected, the CapA322 did not react to vaccinated horse serum (Vac_H3D21), which had a high anti-PAD1 IgG concentration (Fig 2G), indicating that the CapA322 is specific to antibodies resulting from virulent *B*. *anthracis* infection. In addition, its substantial soluble expression confirms that CapA322 is a potent antigen candidate for developing a diagnostic tool.

**Fig 3:**
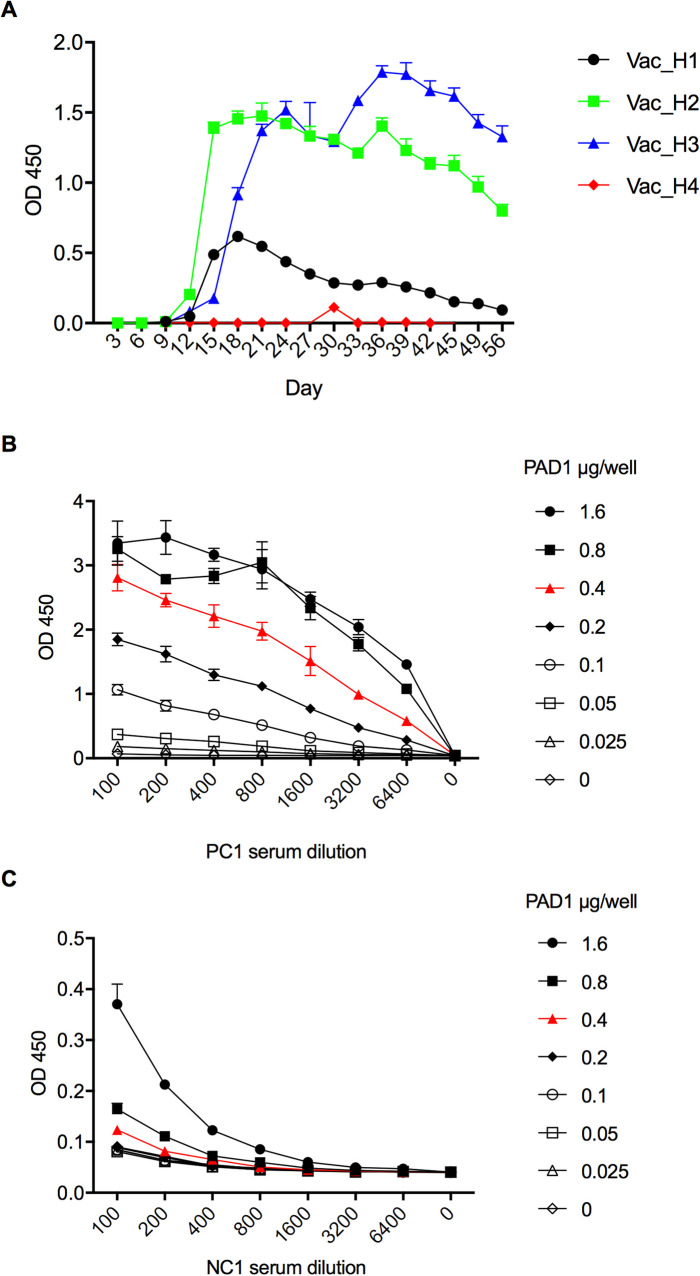
Horses anti-PAD1 immunoglobulin G (IgG) responses against subcutaneously injected *Bacillus anthracis* Sterne 34F2 strain spore vaccine. (A) anti-PAD1 IgG response of vaccinated horses in serum dilution 1:100. Each serum sample was tested in technical triplicate by PAD1-ELISA. Checkerboard titration between PAD1 and (B) horse hyperimmunized anti-*B*. *anthracis* serum (PC1) or (C) naive horse serum (NC1) in PAD1-ELISA. Each dilution of serum sample was tested in technical triplicate. The twofold serum dilution starts with a dilution of 1:100, and PAD1 dilution starts with 1.6 μg/well. From the result, we determined the optimal concentrations of the antigen, antibody, and serum dilutions as follows: antigen, 0.4 μg/well; serum dilution, 1:100; second antibody dilution, 1:15,000.

Further, to elucidate whether CapA322 is cross-reactive to vaccinated horse serum or not, we prepared serum samples from four horses vaccinated with the Sterne vaccine and evaluated the immune responses using a PAD1-ELISA (Fig 3A). PA-D1, domain one of the PA of *B*. *anthracis*, was prepared as previously described [27]. The optimal titration condition of the PAD1-ELISA for a horse was determined as the PA-D1 antigen concentration of 0.4 μg/ml, serum dilution of 1:100, and a secondary antibody dilution of 15,000 (Fig 3B and 3C). The Sterne vaccine elicited immune responses in three horses out of four starting at 15 days postvaccination. Despite being inoculated with the same dose of vaccine, horses 2 (Vac_H2) and 3 (Vac_H3) showed higher antibody responses than horses 1 (Vac_H1) and 4 (Vac_H4) at all time points. The antibody titers in horses 2 and 3 declined only after the second peak around 5 weeks postvaccination. In contrast, horse 1 showed a weak immune response to the vaccination, with titers decreasing soon after the initial peak. However, horse 4 did not show a detectible immune response despite receiving the same dose of vaccine (Fig 3A).

### CapA322-ELISA and PAD1-ELISA

**Fig 4:**
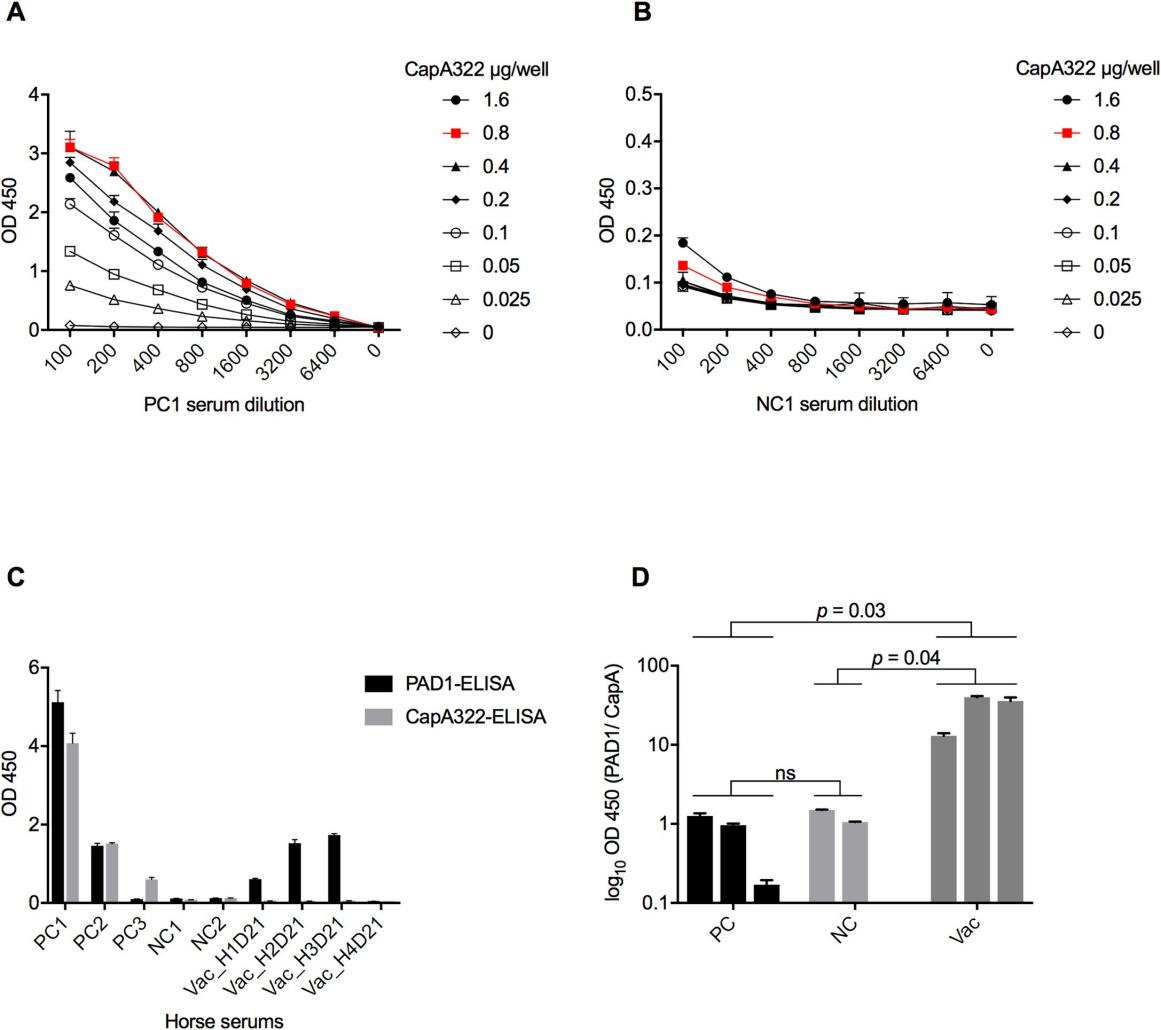
Comparison of PAD1-ELISA and CapA322-ELISA. Checkerboard titration between CapA322 and (A) horse hyperimmunized anti-*Bacillus anthracis* serum (PC1) or (B) naive horse serum (NC1) in CapA322-ELISA. Each dilution of serum sample was tested in technical triplicate by CapA322-ELISA. The twofold serum dilution starts with a dilution of 1:100, and CapA322 dilution starts with 1.6 μg/well. From the result, we determined the optimal concentrations of antigen, antibody, and serum dilutions as follows: antigen, 0.8 μg/well; serum dilution, 1:100; second antibody dilution, 1:15,000. (C) Comparison of PAD1-ELISA and CapA322-ELISA on horse sera. Each serum samples of horse hyperimmunized anti-*Bacillus anthracis* (PC1), horse naturally infected with *B*. *anthracis* (PC2 and PC3), naive horses (NC1 and NC2), and horse vaccinated with Sterne34F2 strain (Vac_H1D21-Vac_H4D21) was analyzed in technical triplicate by PAD1-ELISA and CapA322-ELISA. (D) One-way ANOVA with Tukey’s multiple comparison test on relative OD values of positive (PC; n = 3), negative (NC; n = 2), and vaccinated (Vac; n = 3) groups.

Based on the CapA322 antigen, we developed the CapA322-ELISA. An antigen concentration of 0.8 μg/ml, serum dilution of 1:100, and a secondary antibody dilution of 15,000 were determined as the optimal titration conditions of CapA322-ELISA for horses (Fig 4A and 4B). Each serum sample from horses experimentally (PC1) or naturally infected with virulent *B*. *anthracis* (PC2 and PC3) and horses immunized with Sterne vaccine (Vac_H1D21-Vac_H4D21) as well as naive horses (NC1 and NC2) was technically triplicated and comparatively tested by the CapA322-ELISA and PAD1-ELISA (Fig 4C). As expected, PC1 and PC2 gave a significantly higher OD absorbance than the NC1 and NC2 in both CapA322 and PAD1-ELISAs. Vaccinated horse sera Vac_H1D21–Vac_H3D21 showed high OD absorbance in PAD1-ELISA, whereas all were negative in CapA322-ELISA. However, the PC3 serum exhibited a positive reaction only with CapA322-ELISA but was negative in PAD1-ELISA.

Furthermore, except one sample (PC3), relative OD values in group PC were around 1, suggesting that the two tests (PAD1-ELISA and CapA322-ELISA) were comparable in detecting virulent *B*. *anthracis* infection. Similarly, relative OD values in group NC were around 1, indicating that the two tests give the same result. However, relative OD values in group Vac ranged from 13.0 to 39.9, showing the non-cross reactivity of CapA322-ELISA with vaccine-induced antibodies. This result was supported by the observation that the relative OD values in vaccinated group were significantly higher than those in the positive (*p* = 0.03) and negative (*p* = 0.04) groups (Fig 4D).

## Discussion

In this study, potential antigen candidates encoded by pXO2 were screened by *in silico* analyses, and the CapA322-ELISA was developed to detect antibodies against CapA, which is secreted from virulent *B*. *anthracis* strains that possess pXO2.

Identifying the *B*. *anthracis* natural infection, specifically sublethal infection in animals, is vital in predicting outbreaks in endemic areas. Additionally, identifying sublethal infection can also aid in describing anthrax dynamics in ecosystems and understand the host-pathogen interaction, which is still poorly understood [40]. Current conventional serological assays for anthrax diagnosis and evaluation of immune responses to anthrax vaccines have been developed based on the PA of *B*. *anthracis*, which is reactive to both naturally acquired and vaccine-generated antibodies. Though animals with natural infections could be distinguished from vaccinated animals based on vaccination history, collecting such data is somewhat cumbersome due to data accessibility and missing records. Besides, although the duration of solid immunity among vaccinated animals is no longer than a year [25], a previous study found that residual antibodies in vaccinated animals were still significantly higher than non-vaccinated animals even after a year had passed [27]. Given the importance of serodiagnosis for distinguishing *B*. *anthracis* natural infection from vaccine immunity, we developed the CapA322-ELISA that detects antibodies against CapA encoded by pXO2 of *B*. *anthracis* without cross-reacting with sera from vaccinated animals, shown in Figs 2G and 4C.

Except for a few unusual *B*. *cereus* strains (Table 4), the C-terminus region of CapA, named CapA322, has lower sequence similarity with other bacterial species and showed immunoreactivity with sera from horses infected with virulent *B*. *anthracis*. The γ-D-PGA capsule of *B*. *anthracis*, a critical virulence determinant of *B*. *anthracis*, is synthesized by CapB and CapC, and transported by CapA and CapE across the cell membrane [41]. Thus, CapA is essential for transporting the γ-D-PGA, but the immunoreactivity of this membrane protein is yet to be determined. To the best of our knowledge, this study is the first to identify CapA as immunoreactive. While the whole length of CapA as well as its C-terminal region (CapA322) are immunoreactive (Fig 2C), we selected the CapA322 for ELISA development because it was more soluble (S2 Fig) compared to the whole length protein (Fig 1A and S1 Fig).

CapA is a single-pass transmembrane protein that belongs to the metallophosphatase superfamily [42]. A single transmembrane helix domain is located at the N-terminus region from 25 to 44 of CapA, translocating the protein in the cell membrane, thereby exposing the C-terminus region to the cell outside. This exposed cell surface can define the immunoreactive characteristic of the CapA322. Many antigens or vaccine candidate-searching studies have been targeting surface-exposed proteins [43–45]. Cell surface molecules have a greater chance to generate a host cell immune response by its position as they are more exposed to host cells [46]. Previously, a few unusual *B*. *cereus* strains have been reported to cause anthrax-like disease in animals and humans due to the acquisition of virulence plasmids that are highly similar to the *B*. *anthracis* virulence plasmids pXO1 and pXO2 [47, 48]. Those *B*. *cereus* strains are divided into two variants; atypical strains such as 03BB102 and 03BB108 and *B*. *cereus* biovar anthracis (Bcbva) strains such as BC-AK and CI [49]. *B*. *anthracis* CapA exhibited relatively high sequence homology with the so-called Bcbva and atypical *B*. *cereus* strains (Table 4); therefore, it is possible for CapA322-ELISA to cross-react with such strains. However, these strains have only been reported in rare cases [50–52]. Also, studies suggest that pXO2 plasmid is not commonly distributed in *B*. *cereus* and *B*. *thuringiensis* strains, which are closely related to *B*. *anthracis* [53, 54]. Moreover, screening of pXO2 ORFs among *B*. *cereus* group strains revealed a restricted distribution of *cap* genes other than in *B*. *anthracis* [55]. Further, despite the presence of *cap* genes, it is doubtful whether *cap* genes in atypical *B*. *cereus* strains 03BB102 and 03BB108 are expressed because there was no capsule expression detected in-vitro conditions where it is normally expressed in *B*. *anthracis* [48, 52]. A part of the plasmid harbored by strain 03BB102 is highly similar to a portion of the *B*. *anthracis* pXO2, but the rest of the sequence is different [49]; therefore, *cap* genes expression might be attenuated in this strain, resulting in the absence of capsule. Although CapA homologs in Bcbva and atypical *B*. *cereus* strains are not expected to significantly confound the result of CapA322-ELISA because of the rarity of such strains in nature, further validation is required to examine the specificity of CapA322-ELISA by assessing possible antibody cross-reactivity.

A comparison of results from CapA322-ELISA and PAD1-ELISA showed that CapA322-ELISA could detect anti-CapA antibodies in the sera of horses experimentally and naturally infected with virulent *B*. *anthracis* strains. Several ELISAs have been used for serodiagnosis of *B*. *anthracis* infection, including assays for detecting anti-LF [56] and PA [57] IgGs; however, detected antibodies themselves are insufficient to distinguish the natural infection of *B*. *anthracis* from vaccine immunity. Moreover, there is an ELISA for detecting anti-γ-D-PGA IgG of *B*. *anthracis* [58]. However, Chen Z *et al*. found γ-D-PGA antibodies in sera of *B*. *anthracis* non-infected chimpanzees (n = 9) and humans (n = 6), which were likely the result of exposure to other *Bacillus* species [59]. In our study, anti-CapA antibodies were not detected in any horse sera (n = 6) other than positive samples (n = 3) when the starting dilution of 1:100 was used (Fig 4C). In addition, unlike PA-based ELISA, CapA322-ELISA showed an advantage of non-cross-reactivity with vaccinated horse sera, suggesting that the CapA322 can be a helpful tool for determining the naturally acquired immune response of animals. For further validation of the CapA322-ELISA, screening a larger sample size for horses, including cattle, is needed. Future work will therefore include follow-up work designed to validate the assay.

In preparing vaccinated horse serum samples, we observed significant diversity in the antibody response (Fig 3A). This is in line with data observed by Phaswana *et al*. [60] where five individual Boer goats vaccinated with Sterne 34F2 *B*. *anthracis* strain showed variable levels of immune response. The Sterne vaccine elicited an immune response in three out of four vaccinated horses in our study. However, the anti-PA IgG titers progressively declined from around 5 weeks postvaccination (Fig 3A). This could explain the negative result observed when the serum of PC3 collected from *B*. *anthracis* naturally infected horse was tested by PAD1-ELISA (Fig 4C). Although we could not determine the time interval from infection to sample collection, we speculate that anti-PA IgG titer in PC3 serum had already decreased below detectible levels by that time. Also, as a major limitation of this study, we could not evaluate weekly anti-PA IgG titers in PC2 and PC3 as these were collected at a single point in time and analyzed retrospectively. Nevertheless, the anti-CapA antibodies were detectable by our developed assay, suggesting better stability relative to anti-PA IgG. Although we carefully selected the horses of the same age and sex and subcutaneously vaccinated them with the same lot of vaccine using the same procedure, one horse did not show any immune response (Fig 3A). Therefore, rather than technical vaccination failure, this observation may have been due to differences in the horse’s immunological state or genetic background. Therefore, more studies with various vaccine doses and challenging tasks will be needed to determine the optimal amount for horse vaccination.

In addition to the CapA (GBAA_RS28240), we identified peptide ABC transporter substrate-binding protein (GBAA_RS28340) as immunoreactive. The peptide ABC transporter substrate-binding protein was previously reported to be seroreactive with antisera of rabbit and mice infected with *B*. *anthracis* Ames spore, and convalescent serum from rhesus macaques vaccinated with Anthrax Vaccine Adsorbed (AVA, BioThrax) [61]. Chitlaru T *et al*. [62] highlighted that substrate-binding proteins of ABC transporters are highly immunogenic protein classes. It also noteworthy that Orit Gat *et al*. conducted a similar study in search of potential immunogen proteins from the *B*. *anthracis* genome, including two virulence plasmids. Three of their eight selected proteins from pXO2, lysozyme (GBAA_RS28005), amidase (GBAA_RS28165), and metal-dependent hydrolase (GBAA_RS28430), coincided with our selection. Although we failed to clone CDSs of lysozyme (GBAA_RS28005) and amidase (GBAA_RS28165), they determined that these two proteins are immunoreactive with guinea pig and rabbit hyperimmunized with Vollum strain of *B*. *anthracis* (pXO1^+^, pXO2^+^) [63]. In contrast, metal-dependent hydrolase (GBAA_RS28430) was negative, which was the same as our result. The immunoreactive proteins identified here and in previous studies [36, 61–64] may be the first candidates for future diagnostic developments. These proteins may provide valuable additives for AVA improvement; the vaccine is currently licensed for humans and consists primarily of PA [65]. Although the vaccine is effective, expanding the vaccine protection by including additional antigens in its formulation is necessary for a less demanding vaccination regimen and as a defense against bioterrorism [66]. Further, regardless of the protective immunity provided by PA, previous studies noted that relying on antibodies against PA as the sole protector against anthrax is unconvincing due to the variable protection level conferred by antibodies generated by the PA-based vaccine [67–70]. Our study showed the possibility of antibodies against PA being short-lived in the sera of vaccinated animals, which declined to range from 3 to 5 weeks after immunization (Fig 3A). Considering these aspects, adding recombinant antigens to the PA might increase the durability and protective efficacy of the AVA vaccine with a less demanding vaccination regimen through a multi-antigen cocktail vaccine, as has been achieved against *Bordetella pertussis* infection [71].

## Conclusion

Screening of potential ELISA antigen candidate CDSs in the *B*. *anthracis* virulence pXO2 was conducted through *in silico* and immunoreactivity analyses. The capsule biosynthesis protein CapA and peptide ABC transporter substrate-binding protein are identified as immunoreactive. Concerning the antigen specificity, immunoreactivity, and solubility of protein expression, the C-terminus end of capsule biosynthesis protein CapA, identified in this study and named CapA322, was further selected and used for CapA322-ELISA development. The CapA322-ELISA was shown to be specific and non-cross-reactive to sera from horses vaccinated with *B*. *anthracis* Sterne 34F2 strain live spore vaccine. Hence, the CapA322-ELISA can be used to detect naturally acquired antibodies and ascertain the immunological state of animals. Continued research is essential to optimize the CapA322-ELISA. Future work will involve field studies of livestock in endemic and non-endemic areas to validate the assay for target animal species.

## Supporting information

S1 Fig Expression of candidate proteins. (A–E) Coomassie brilliant blue (CBB) staining analyses of proteins in cell lysate (lanes 2–5), supernatant (lanes 6–9), and pellet (lanes 10–13) fractions of control and candidate protein-expressing *Escherichia coli* strain grown in terrific broth with or without isopropyl β-D-thiogalactopyranoside (IPTG) at 37°C for 4 h at 180 rpm. Lanes 1 and 14, Mw, molecular weight marker (in kDa). Control, *E*. *coli* BL21 harboring empty pGEX-6P-2 plasmid expressing glutathione S-transferase (GST: 26 kDa). (A) *E*. *coli* BTZ001 expressing recombinant hypothetical protein (GST-GBAA_RS28110: 44 kDa). (B) *E*. *coli* BTZ002 expressing recombinant capsule biosynthesis protein CapA (GST-GBAA_RS28240: 72 kDa). (C) *E*. *coli* BTZ003 expressing recombinant signal peptidase (GST-GBAA_RS28275: 47 kDa). (D) *E*. *coli* BTZ004 expressing recombinant peptide ABC substrate-binding protein (GST-GBAA_RS28340: 84 kDa). (E) *E*. *coli* BTZ005 expressing recombinant metal-dependent hydrolase (GST-GBAA_RS28430: 46 kDa). +, 0.2 mM IPTG induction; −, without IPTG induction.
(TIF)

S2 Fig Expression of CapA322. (A and B) Coomassie brilliant blue staining (CBB) and Western blotting of proteins in cell lysate (lanes 2–5), supernatant (lanes 6–9), and pellet (lanes 10–13) fractions of control and CapA322 expressing *Escherichia coli* BTZ006 grown in terrific broth with or without isopropyl β-D-thiogalactopyranoside (IPTG) at 37°C for 4 h at 180 rpm. In Western blotting, the proteins were probed with anti-glutathione S-transferase (GST) immunoglobulin G. Lanes 1 and 14, Mw, molecular weight marker (in kDa). Control, *E*. *coli* BL21 harboring empty pGEX-6P-2 plasmid expressing GST (GST: 26 kDa). BTZ006, *E*. *coli* expressing recombinant CapA322 (GST-CapA322: 37 kDa). +, 0.2 mM IPTG induction; −, without IPTG induction.
(TIF)

S1 Table Result of *in silico* analyses on CDSs encoded by pXO2. (XLSX)

S1 Raw images (PDF)
