# Supplementary figures and images for: Development of ELISA based on Bacillus anthracis capsule biosynthesis protein CapA for naturally acquired antibodies against anthrax
Source: PLoS One. 2021 Oct 11;16(10):e0258317. doi: 10.1371/journal.pone.0258317 (PMC8504768; doi:10.1371/journal.pone.0258317)

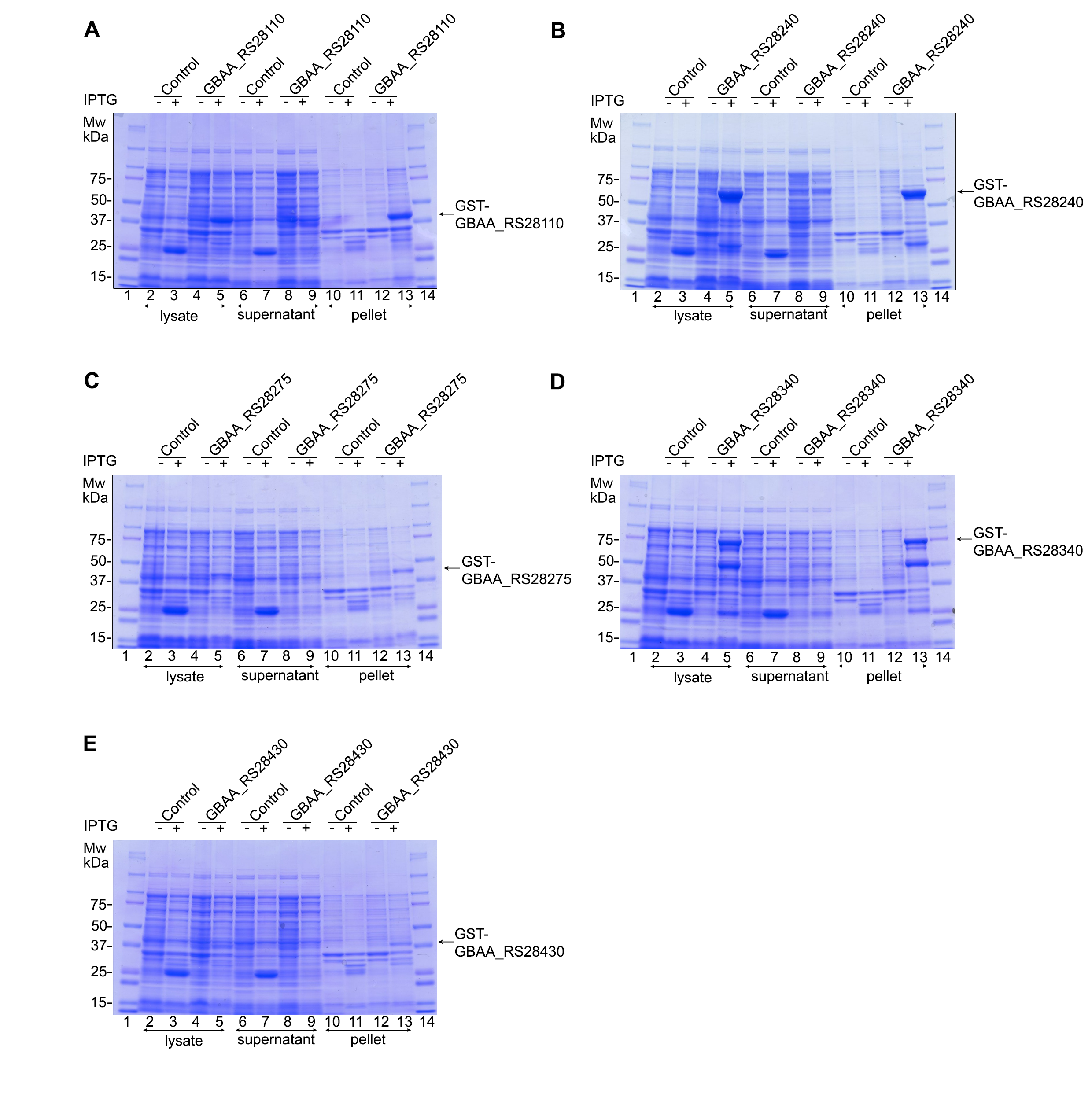

Supplement: S1 Fig — (A–E) Coomassie brilliant blue (CBB) staining analyses of proteins in cell lysate (lanes 2–5), supernatant (lanes 6–9), and pellet (lanes 10–13) fractions of control and candidate protein-expressing Escherichia coli strain grown in terrific broth with or without isopropyl β-D-thiogalactopyranoside (IPTG) at 37°C for 4 h at 180 rpm. Lanes 1 and 14, Mw, molecular weight marker (in kDa). Control, E. coli BL21 harboring empty pGEX-6P-2 plasmid expressing glutathione S-transferase (GST: 26 kDa). (A) E. coli BTZ001 expressing recombinant hypothetical protein (GST-GBAA_RS28110: 44 kDa). (B) E. coli BTZ002 expressing recombinant capsule biosynthesis protein CapA (GST-GBAA_RS28240: 72 kDa). (C) E. coli BTZ003 expressing recombinant signal peptidase (GST-GBAA_RS28275: 47 kDa). (D) E. coli BTZ004 expressing recombinant peptide ABC substrate-binding protein (GST-GBAA_RS28340: 84 kDa). (E) E. coli BTZ005 expressing recombinant metal-dependent hydrolase (GST-GBAA_RS28430: 46 kDa). +, 0.2 mM IPTG induction; −, without IPTG induction. (TIF) [file pone.0258317.s001.tif]

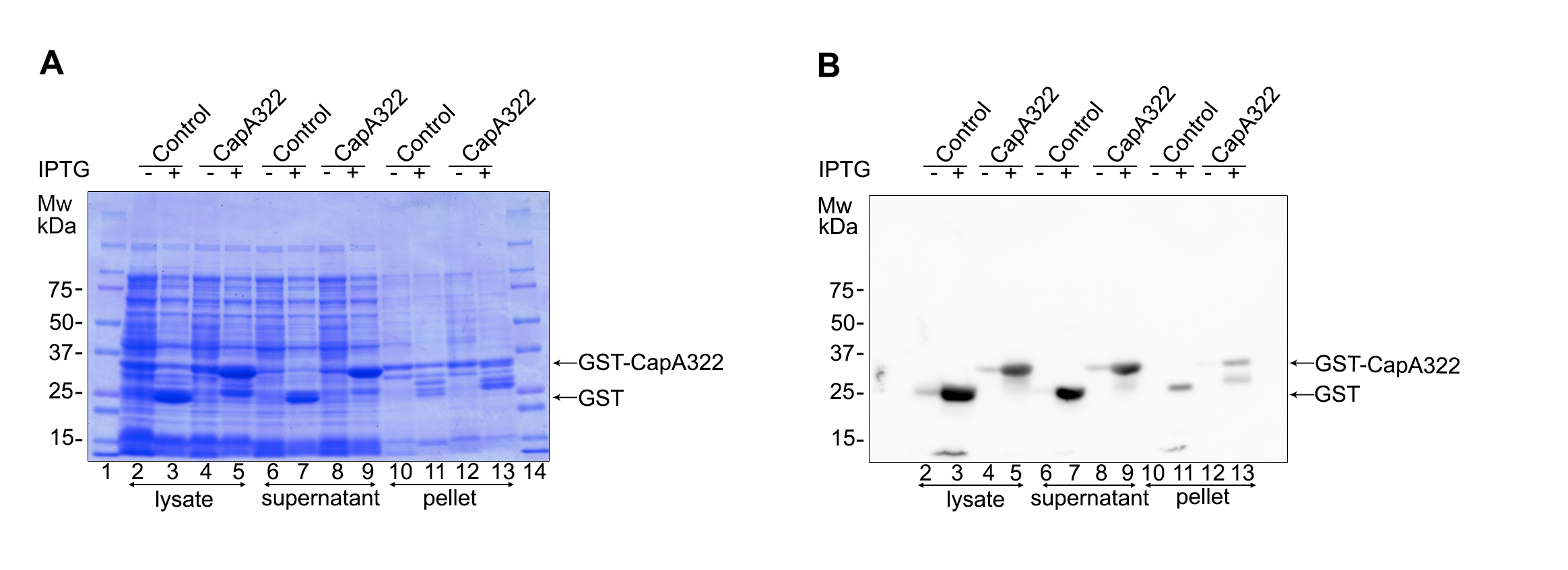

Supplement: S2 Fig — (A and B) Coomassie brilliant blue staining (CBB) and Western blotting of proteins in cell lysate (lanes 2–5), supernatant (lanes 6–9), and pellet (lanes 10–13) fractions of control and CapA322 expressing Escherichia coli BTZ006 grown in terrific broth with or without isopropyl β-D-thiogalactopyranoside (IPTG) at 37°C for 4 h at 180 rpm. In Western blotting, the proteins were probed with anti-glutathione S-transferase (GST) immunoglobulin G. Lanes 1 and 14, Mw, molecular weight marker (in kDa). Control, E. coli BL21 harboring empty pGEX-6P-2 plasmid expressing GST (GST: 26 kDa). BTZ006, E. coli expressing recombinant CapA322 (GST-CapA322: 37 kDa). +, 0.2 mM IPTG induction; −, without IPTG induction. (TIF) [file pone.0258317.s002.tif]
