# Supplementary material for: Development of ELISA based on Bacillus anthracis capsule biosynthesis protein CapA for naturally acquired antibodies against anthrax
Source: PLoS One. 2021 Oct 11;16(10):e0258317. doi: 10.1371/journal.pone.0258317 (PMC8504768; doi:10.1371/journal.pone.0258317)
Supplement: S1 Raw images — (PDF) [file pone.0258317.s004.pdf]

A

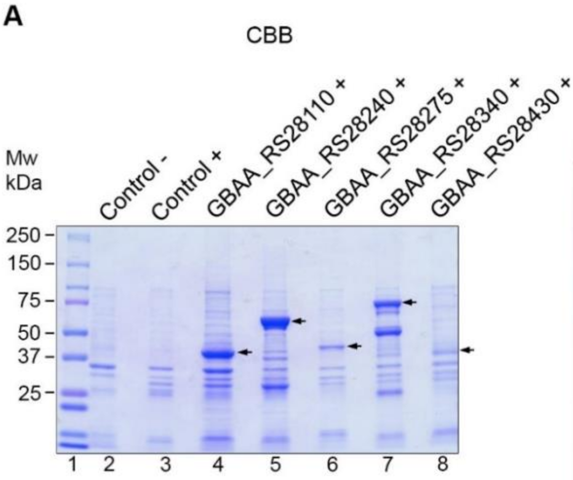
CBB
Mw kDa
Control -
Control +
GBAA_RS28110 +
GBAA_RS28240 +
GBAA_RS28275 +
GBAA_RS28340 +
GBAA_RS28430 +
250
150
75
50
37
25
1 2 3 4 5 6 7 8

A raw

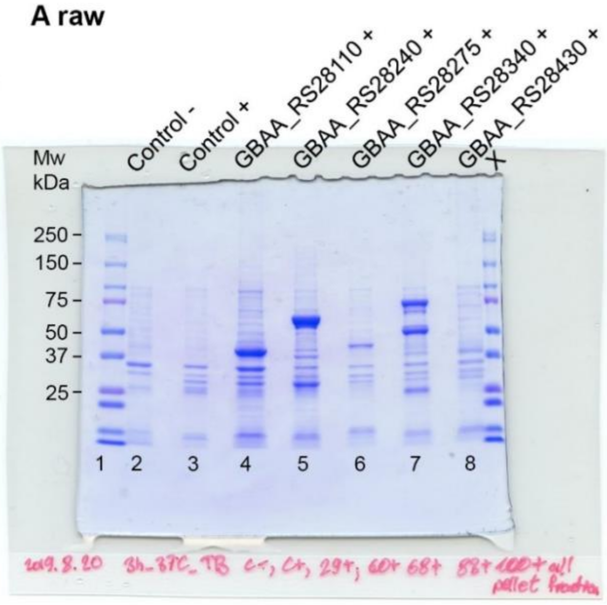
Control -
Control +
GBAA_RS28110 +
GBAA_RS28240 +
GBAA_RS28275 +
GBAA_RS28340 +
GBAA_RS28430 +
Mw kDa
250
150
75
50
37
25
1 2 3 4 5 6 7 8
2019.8.20 3h_37C_TB C-, C+, 29+, 60+ 68+ 88+ 100+ all pellet fraction

B

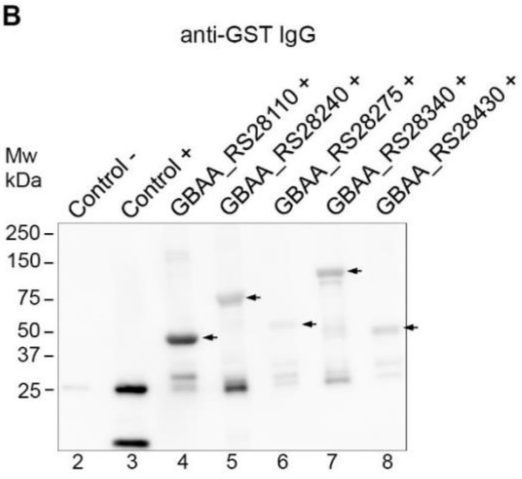
anti-GST IgG
Mw kDa
Control -
Control +
GBAA_RS28110 +
GBAA_RS28240 +
GBAA_RS28275 +
GBAA_RS28340 +
GBAA_RS28430 +
250
150
75
50
37
25
2 3 4 5 6 7 8

B raw

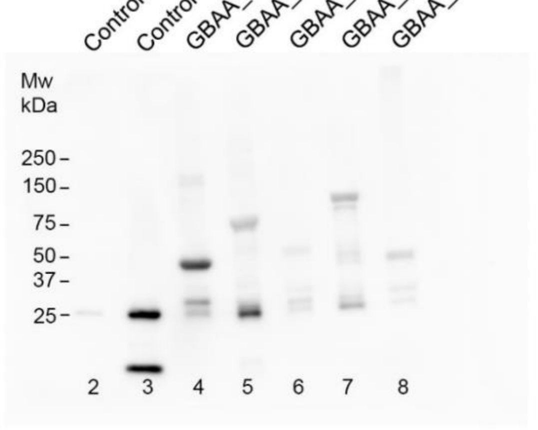
Control -
Control +
GBAA_RS28110 +
GBAA_RS28240 +
GBAA_RS28275 +
GBAA_RS28340 +
GBAA_RS28430 +
Mw kDa
250
150
75
50
37
25
2 3 4 5 6 7 8

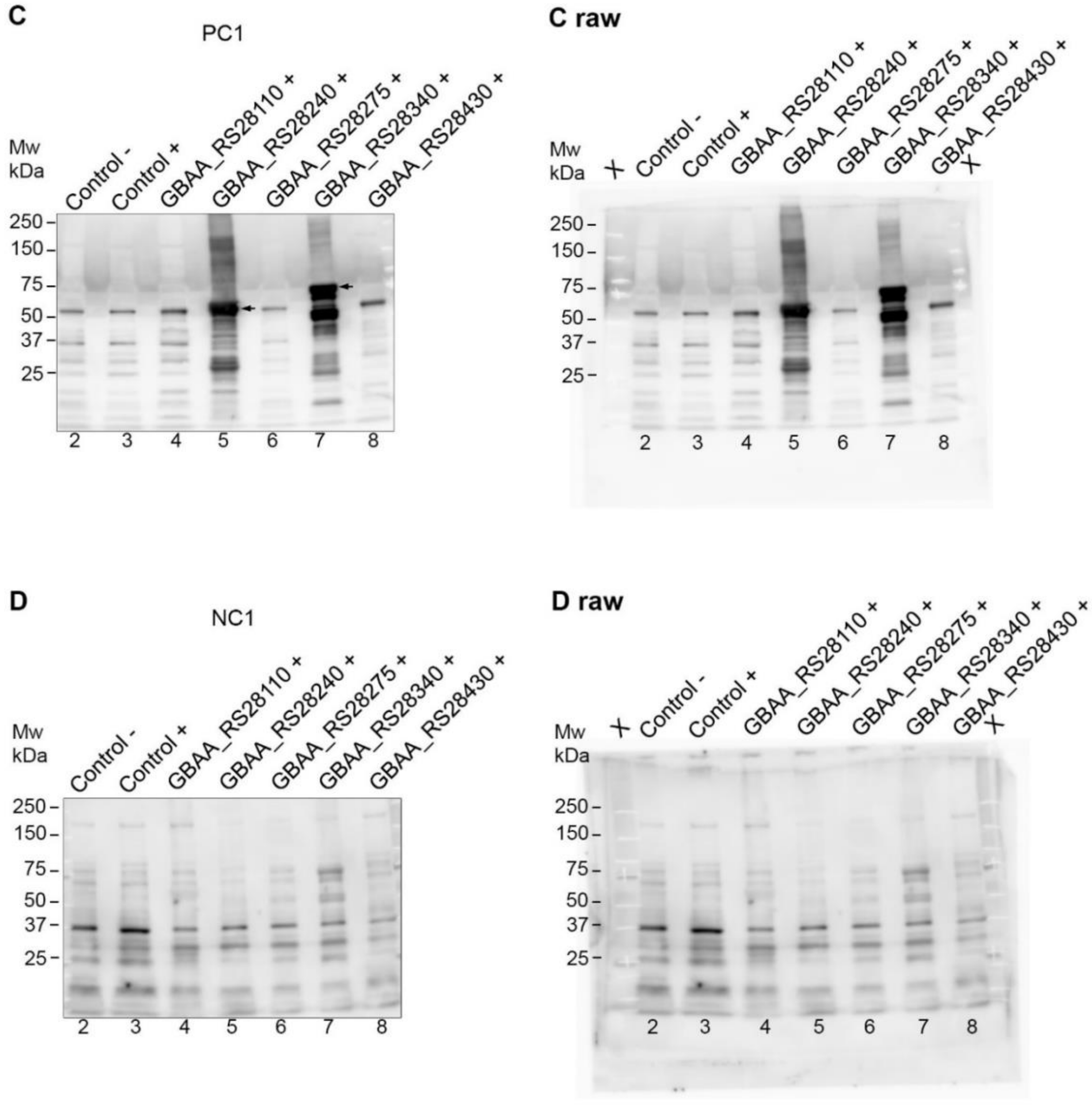

Fig 1. Adjusted figures are on the left, and their raw images are on the right. Gel Images were captured by EPSON GT-X970 scanner. Blot images were captured by ImageQuant LAS 4000 (GE Healthcare).

B

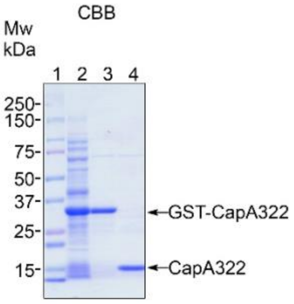

B raw

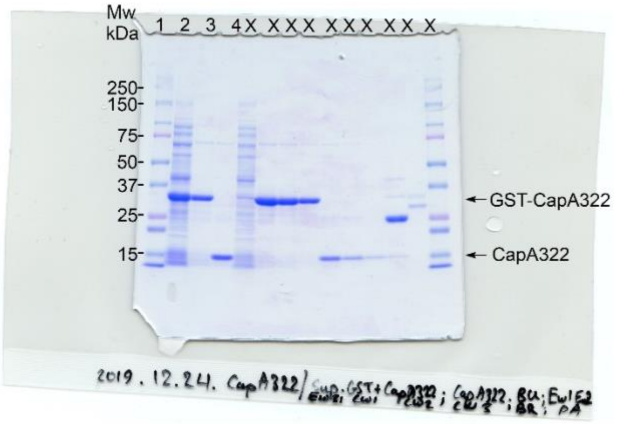

C

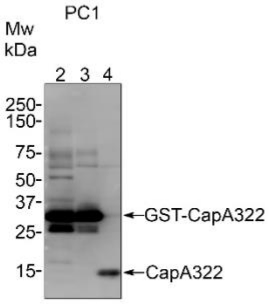

C raw

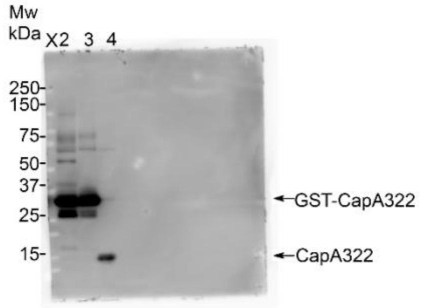

D

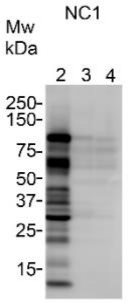

D raw

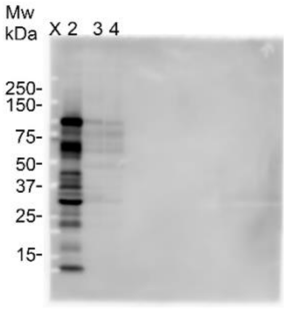

E

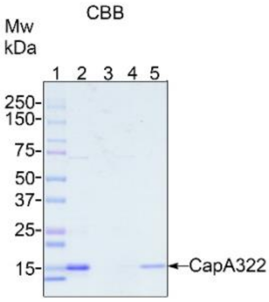

E raw

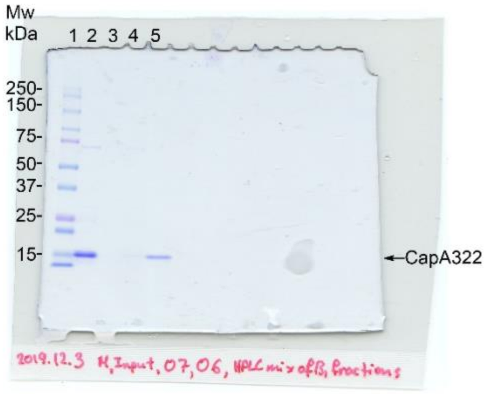

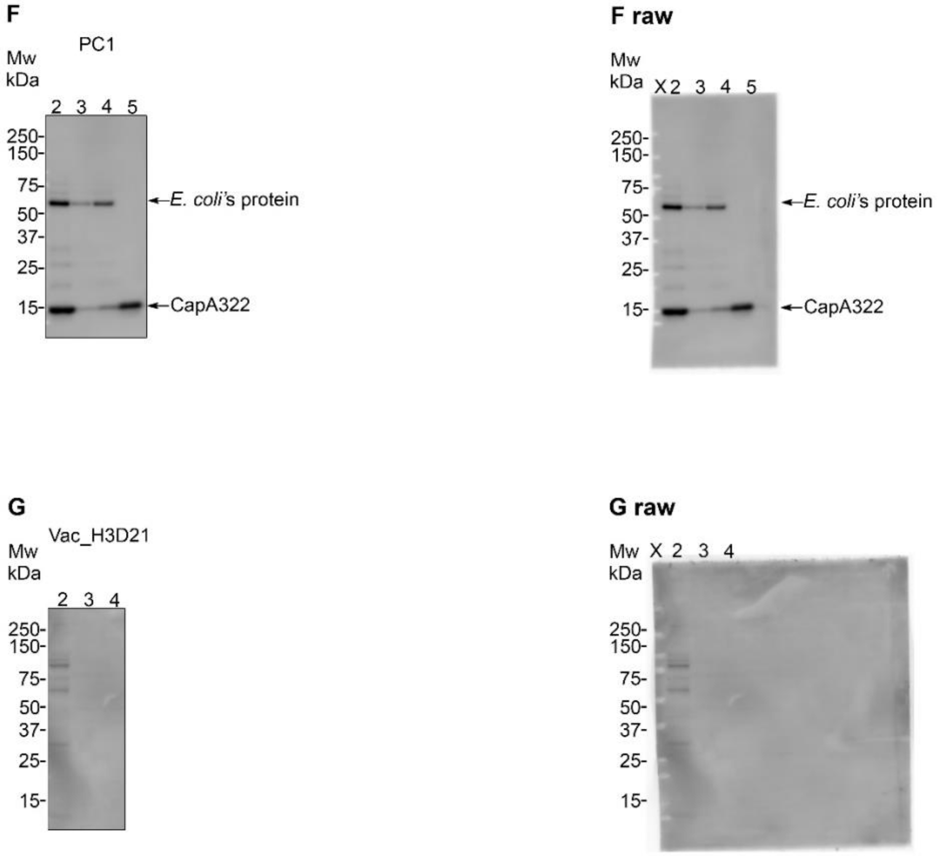

Fig 2. Adjusted figures are on the left, and their raw images are on the right. Gel Images were captured by EPSON GT-X970 scanner. Blot images were captured by ImageQuant LAS 4000 (GE Healthcare).

A

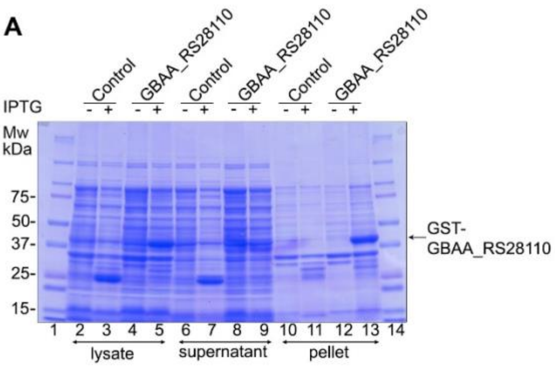

A raw

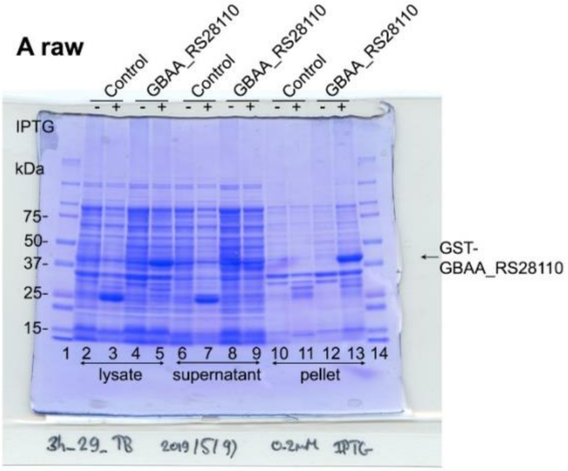

B

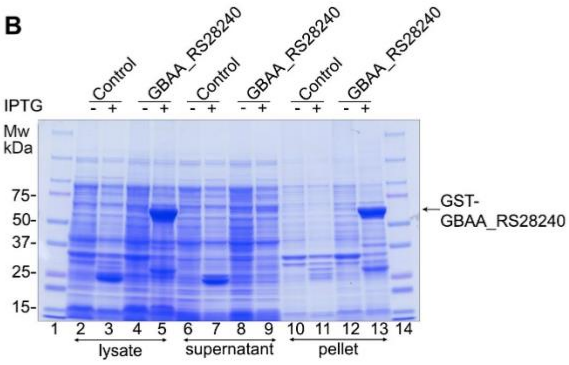

B raw

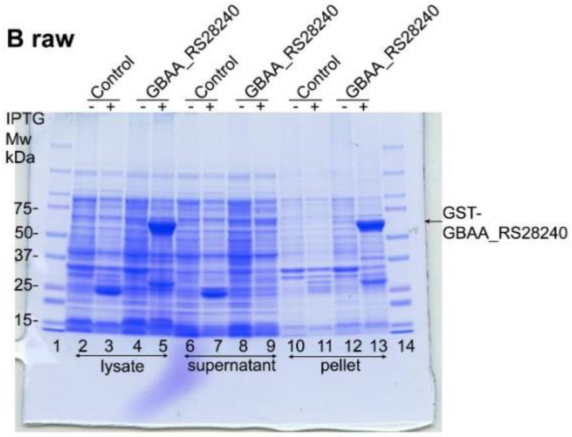

C

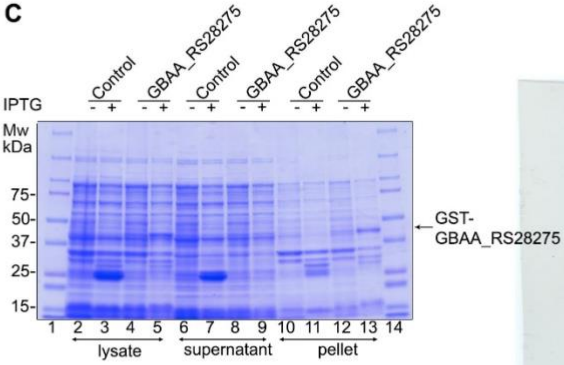

C raw

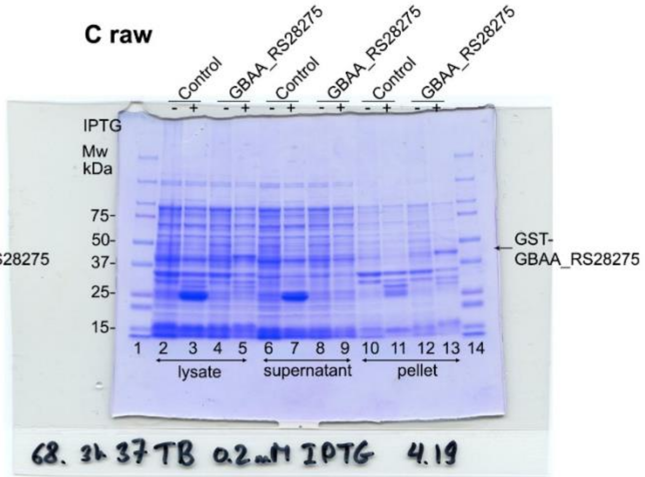

D

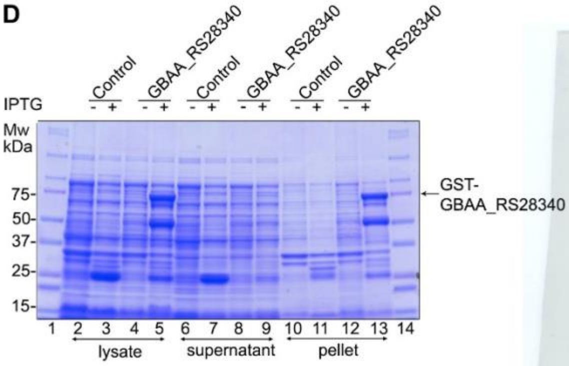

D raw

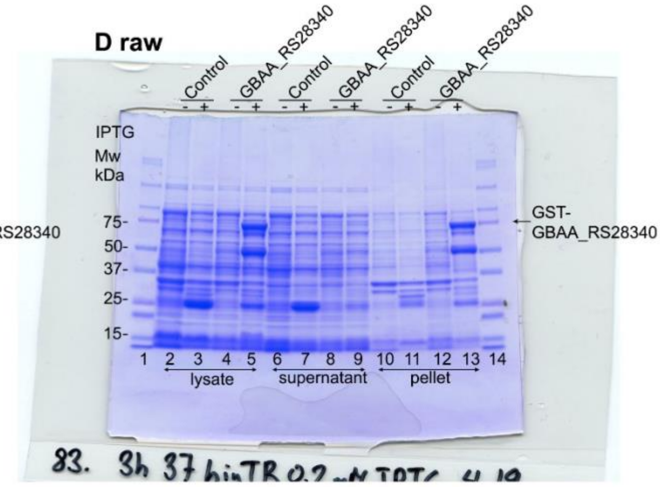

E

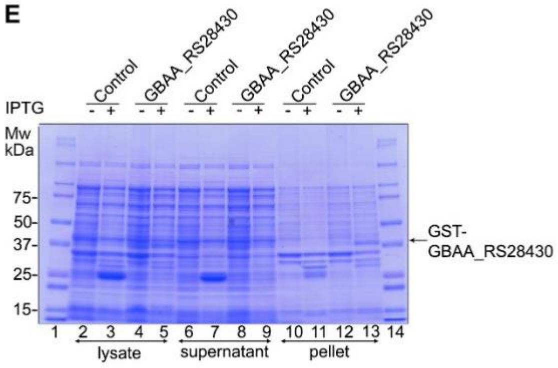

E raw

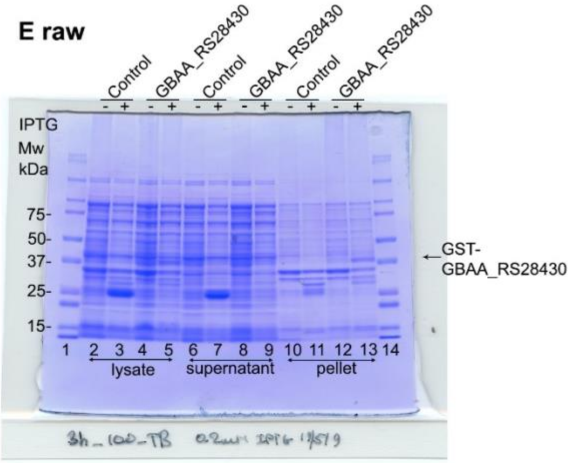

S1 Fig. Adjusted figures are on the left, and their raw images are on the right. Gel Images were captured by EPSON GT-X970 scanner.

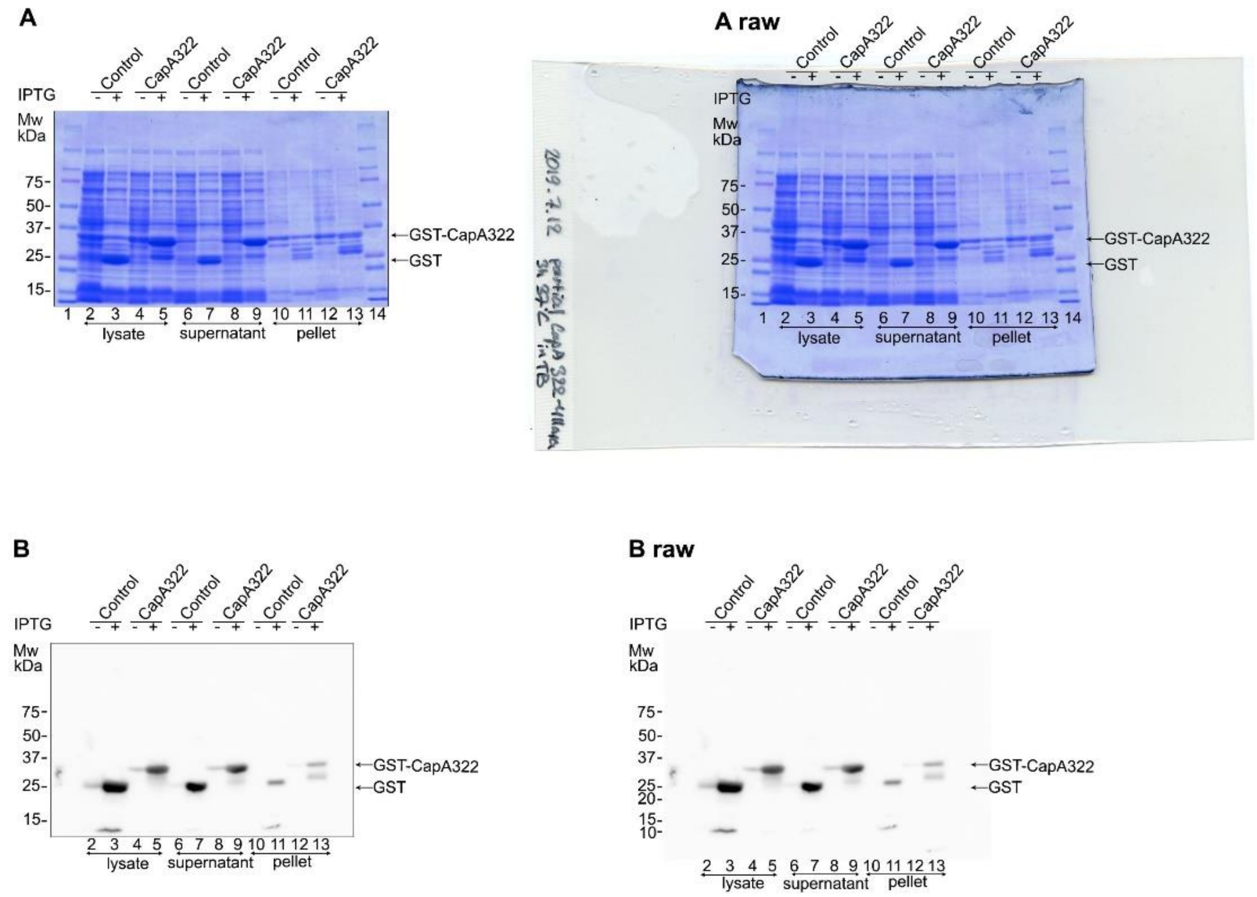

S2 Fig. Adjusted figures are on the left, and their raw images are on the right. Gel Images were captured by EPSON GT-X970 scanner. Blot images were captured by ImageQuant LAS 4000 (GE Healthcare).
